# Adolescents’ Experiences with Being Weighed at School: A Qualitative Interview Study in Norway

**DOI:** 10.1177/23333936261435414

**Published:** 2026-03-27

**Authors:** Marit Kjesbo Risøy, Maria Gangdal Gunnarskog

**Affiliations:** 1 Western Norway University of Applied Sciences, Bergen, Norway

**Keywords:** public health nursing, school-based weighing, qualitative research, adolescents, Body Mass Index (BMI) screening, Norway

## Abstract

School-based weight measurements are routinely used in public health surveillance and are intended to support the early identification of health risks. However, concerns have been raised regarding unintended consequences, and research on pupils’ experiences remains limited. This study explored how upper secondary school pupils retrospectively reflect on being weighed in school. Semi-structured interviews were conducted with 16 adolescents aged 16 to 19 years in Western Norway, and the data were analyzed using systematic text condensation. Participants described responses ranging from neutrality to increased body awareness, body dissatisfaction, and, in some cases, the onset of eating disorders. Weighing can prompt peer discussions, including pressure to disclose one’s weight and to engage in body comparisons. Organizational factors such as limited privacy, lack of information, and unclear voluntariness shaped experiences that could undermine the intended health-promoting purpose. These findings indicate that school-based weighting poses psychosocial risks that undermine its health-promoting aims and contribute to the broader debate over whether such practices should continue. To better align with health-promoting goals, school health services should ensure privacy, provide clear communication about the purpose and voluntariness of weighing, and offer opportunities for dialogue. Public health nurses should use sensitive and stigma-free communication when weight deviations are identified.

## Introduction

Overweight and obesity among children and adolescents represent growing global public health challenges (Abarca-Gómez et al., 2017; WHO, 2016). Globally, the prevalence of overweight or obesity in children and adolescents increased from 8% in 1990 to 20% in 2022 (WHO, 2025). In Norway, approximately 17% of children are overweight, and 3% to 4% are obese (NIPH, 2024; Øvrebø et al., 2021). In line with the WHO recommendations, Norway and several other countries have introduced Body Mass Index (BMI) measurements in schools as a targeted public health intervention (Branca et al., 2007; The Norwegian Directorate of Health, 2017; WHO, 2017). These measures play a critical role in public health surveillance (Branca et al., 2007; WHO, 2016, 2017). At the individual level, they offer a standardized approach to assessing a child’s health status, facilitating the early identification of weight-related deviations (The Norwegian Directorate of Health, 2010, 2017; WHO, 2016, 2017).

In Norway, national guidelines state that BMI measurements should be conducted by public health nurses (PHNs) in grades 1 (age 6), 3 (age 8), and 8 (age 13) as part of routine school health consultations (The Norwegian Directorate of Health, 2017). These consultations address topics such as mental health, well-being, nutrition, sleep, and physical activity. Measurements should be conducted privately and clearly explained to both pupils and parents in advance. If deviations in weight or height are identified, the results shall be communicated to parents in a timely and comprehensible manner, along with relevant follow-up options (The Norwegian Directorate of Health, 2017).

PHNs play a central role in school health services within a preventive and health-promoting framework (Andersen et al., 2022). While the intention behind weight screening is to prevent unhealthy weight development, some concerns have been raised from PHNs about unintended consequences, including emotional strain, body dissatisfaction, limited resources and follow-up for children with weight deviations (Helseth et al., 2017; Nordstrand et al., 2016; Nygaard & Øen, 2024). These concerns align with a growing body of international literature suggesting that school-based weight practices may unintentionally contribute to psychological distress, body dissatisfaction, and experiences of weight stigma (Crawford et al., 2011; Henningsen et al., 2015; Ikeda et al., 2006; Ramos Salas, 2015; Soto & White, 2010; Thompson & Madsen, 2017).

Body dissatisfaction is prevalent among children and adolescents and is closely linked to self-perceived BMI (Dion et al., 2015, 2016; Martini et al., 2023; Tatangelo et al., 2016). From a public health perspective, this is concerning, as body dissatisfaction is associated with several adverse outcomes, including an increased risk of disordered eating (Bornioli et al., 2019; Lantz et al., 2018; Munkholm et al., 2016) self-harm and substance use (Bornioli et al., 2019). Weight-focused public health strategies for obesity have been criticized as ineffective and potentially amplifying stigma and body dissatisfaction (Puhl & Heuer, 2010; Robinson et al., 2017; Salas, 2015). Weight stigma has been linked to negative outcomes, such as low self-esteem, reduced help-seeking, and weight gain among individuals with higher body weight (Puhl & Suh, 2015; Rubino et al., 2020).

Despite these concerns and the widespread implementation of BMI measurements, few studies have investigated how pupils experience this practice (Altman et al., 2022; Drilen et al., 2024; Igler et al., 2022; Jessen et al., 2023; Madsen et al., 2021; Nnyanzi, 2016). In a recent Norwegian cross-sectional study (*n* = 209), only 1% of 8 to 9-year-olds reported dissatisfaction with the measurements (Drilen et al., 2024). In a larger U.S. cross-sectional study (*n* = 11,510), 64% of pupils reported that being weighed at school did not affect them, while 36% reported varying degrees of discomfort (Altman et al., 2022). In a smaller U.S. survey-based study (*n* = 151), 40% reported that the process of being weighed at school was uncomfortable (Igler et al., 2022).

A recent systematic review and a large U.S.-based randomized controlled trial indicated that weighing at school does not improve pupils’ weight outcomes but may contribute to increased body dissatisfaction and more frequent peer discussions about weight (Jessen et al., 2023; Madsen et al., 2021). The only qualitative interview study conducted in the past decade exploring pupils’ perspectives found that school-based weighing increased weight-related worry across weight categories, particularly among pupils identified to have a weight problem (Nnyanzi, 2016). In qualitative survey findings from Igler et al. (2022), 19% of pupils who had previously been weighed at school reported that they consider school weighing policies to be unhelpful, while 14% found them helpful. Igler et al. (2022) also found that a substantial proportion of those who had been weighed at school did not know why the weighing is conducted.

Despite increasing attention to the psychosocial aspects of weight measurement, existing research remains largely survey-based and offers limited insight into pupils’ reflections. To date, no study has explored how adolescents retrospectively interpret their experiences with school-based weight measurements in a qualitative interview study. This lack of qualitative inquiries represents a significant gap in literature.

This study aimed to explore how upper secondary school pupils retrospectively reflected on their earlier experiences with weight measurements at school. By interviewing adolescents who participated in these practices during primary or lower secondary school, the study seeks to generate a deeper understanding of how these encounters are experienced and interpreted over time. This knowledge can provide insight into how PHNs conduct such practices.

## Methods

### Design

This study employed a qualitative descriptive design, inspired by phenomenological principles (Malterud, 2012), to explore how adolescents experience being weighed in school. An inductive approach was employed, allowing insights to emerge from participants’ accounts and to move from individual experiences to broader patterns (Malterud, 2012). Data were analyzed using systematic text condensation (STC), a stepwise, pragmatic method suitable for examining lived experiences across cases (Malterud, 2012). Inspired by phenomenological thinking, the STC does not require adherence to a specific philosophical framework (Malterud, 2012). This method was selected for its structured and pragmatic approach to synthesizing participants’ experiences into analytical descriptions grounded in their narratives.

### Sample and Recruitment

Participants were recruited from seven upper secondary schools across three municipalities in Western Norway, encompassing both urban and rural areas. Recruitment primarily occurred through information stands in schools, which served as the primary strategy for participant inclusion. Additional outreach was conducted through classroom visits and social media platforms (Facebook, Instagram, and Snapchat).

Twelve participants were recruited from the school stands. Ten participants were interviewed on the same day they were recruited. Two participants were recruited through social media, and two through a classroom visit. Four additional pupils initially expressed interest but later withdrew: one did not respond after the initial contact, and three did not attend the scheduled interviews without providing a reason.

A purposive sampling strategy was employed to include participants of diverse genders, ages, and school backgrounds. The intention was to illuminate the research question from multiple perspectives, following the principles of maximum variation sampling (Polit & Beck, 2021). However, the final sample was influenced by who chose to participate and who was available at the time of recruitment; therefore, elements of convenience sampling are also present (Polit & Beck, 2021).

Participants’ weight status was identified based on their interview accounts, in which they reported the feedback they had received from the public health nurse at the time of measurement. Participants’ weight status is described using the categories normal weight, overweight, and underweight. None of the participants reported being classified as having a specific degree of underweight or overweight. Participants were asked to draw on school weighing experiences they could recall. Four participants reported changes in weight status from primary to lower secondary school: one boy from overweight to normal weight, two girls from overweight to underweight, and one boy from underweight to normal weight. Three boys had experiences of being classified as overweight during primary and/or lower secondary school. One boy and three girls had experiences of being classified as underweight during these school years. Three girls and two boys reported experiences of being classified within the normal-weight range during primary and/or lower secondary school.

Participants were eligible for inclusion if they were between 16 and 19 years old and had been weighed during primary or lower secondary school. The final sample consisted of 16 adolescents: eight girls and eight boys. The mean age was 16.4 years, and all participants were fluent in Norwegian. An overview of participants’ educational backgrounds, sex, age, and school area (urban or rural) is presented in Table 1.

**Table 1.** Overview of Participants.

Educational program	Gender	Age	School area
Sales, service, and tourism	Male	17	Rural
Sales, service, and tourism	Male	17	Rural
Health and social care	Male	17	Rural
Health and social care	Female	18	Rural
Health and social care	Female	16	Rural
Specialization in general studies	Female	16	Rural
Specialization in general studies	Female	17	Urban
Specialization in general studies	Female	16	Urban
Information technology	Male	17	Urban
Building and construction	Male	16	Urban
Building and construction	Male	16	Urban
Building and construction	Male	16	Urban
Building and construction	Male	16	Urban
Electrical studies	Female	16	Urban
Electrical studies	Female	16	Urban
Technology and industrial production	Female	16	Urban

### Data Collection

Data were collected through individual semi-structured interviews conducted between October and December 2024. The interview guide included questions addressing adolescents’ experiences of being weighed at school, social reactions, self-perception, the perceived purpose of weight measurements, and the role of PHNs. The interview guide provided topical guidance and was used flexibly, allowing the interviewer to adapt questions in response to participants’ narratives (Supplemental Material).

Health professionals and a youth advisory panel from the Norwegian Organization Counselling on Eating Disorders reviewed it (ROS, 2025). Input from the youth panel was particularly valuable in ensuring that the questions were relevant and understandable to adolescents. The guide was subsequently refined to improve relevance and clarity for the target group.

Both researchers were present during all interviews, which lasted between 20 and 40 min. One researcher led the interviews, while the other managed the audio equipment, took observational notes, and asked follow-up questions. Because the participants were adolescents, the presence of two adults had the potential to reinforce power differentials or cause discomfort. To mitigate this, interview locations were chosen by the participants to ensure comfort and privacy. Most interviews were conducted at the participants’ schools, with one held in the participants’ home and another in a public library. All participants were informed in advance that two researchers would be present and were given a clear explanation of each researcher’s role.

Having both researchers present served an important methodological function. It allowed for richer and more trustworthy data by capturing nuances, nonverbal reactions, contextual details, and potential areas for follow-up that a single interviewer might overlook. This approach also provided opportunities for participants to clarify or elaborate on their responses. Additionally, the involvement of two interviewers facilitated immediate post-interview debriefing, enabling the researchers to compare interpretations and critically reflect on how their preconceptions might have shaped the dialogue.

The interviews were transcribed using automatic speech-to-text software and were subsequently reviewed manually for accuracy. All transcripts were cross-checked against the audio recordings to ensure fidelity to participants’ accounts, resulting in 239 pages of transcribed material.

### Data Analysis

Data were analyzed using Systematic Text Condensation (STC), a four-step descriptive method inspired by phenomenology developed by Malterud (2012), suitable for systematically exploring participants’ experiences.

The analyses were conducted collaboratively by both researchers. Both are public health nurses with 1.5 years of experience in school health services at the time of data collection and analysis, including direct involvement in weighing pupils before and during the study period. This practical experience provided a distinct pre-understanding of the routines, challenges, and professional expectations associated with weight assessment. Reflexive awareness of the researcher’s positions is essential to reduce bias and enhance analytical transparency (Malterud, 2012). This pre-understanding was acknowledged and continually reflected upon throughout the analytical process, to approach the data with openness and attentiveness to participants’ voices.

In the first step, both researchers independently read all the transcripts to gain an overall impression of the data. Efforts were made to reduce the bias of preconceptions and remain open to participants’ perspectives. Each researcher noted preliminary themes, which were then discussed and refined into five overarching themes: (1) weighing could lead to more talk about weight; (2) weighing contributed to a negative body image; (3) weighing could lead to changes in diet and exercise; (4) lack of information, privacy, and follow-up; and (5) clear gender differences in how weighing was experienced.

Second, meaning units relevant to the research question were identified through line-by-line reading and organized into code groups using NVivo software. Third, each code group was divided into subgroups reflecting different aspects of meaning. Condensed first-person narratives were developed for each subgroup to capture the core content while preserving the tone of participants’ language.

In the final step, the condensates were synthesized into five primary analytical categories that structured the findings. As outlined in the STC methodology, the process is iterative and reflexive, involving continuous movement between data and interpretation to ensure analytical depth and transparency (Malterud, 2012). The selected participant quotations were used to illustrate and support the findings. An example of the analytical process is presented in Table 2.

**Table 2.** Example of the Analysis Process.

Meaning unit	Condensed meaning unit	Code	Theme
I didn’t like my body at all. I wished I were much thinner, no matter what I weighed, and I saw my body in a completely different way. So I basically developed a distorted image of myself.	After I was weighed, I started seeing my body in a new way. The number on the scale made me more aware of my appearance. I began noticing details about my body that I hadn’t thought about before.	A change in body image	From neutral reactions to heightened body awareness
I thought I was fat, that I was ten times bigger than everyone else, and that I didn’t really deserve food because I was so big. It was really just the whole situation, and I’ve never liked the weighing situation either, so all of this has, in the end, given me an eating disorder.	Weighing at school triggered a lot of thoughts, feelings, and changes in how I relate to my body, food, and exercise. I started thinking I was too big, and at times I barely ate or tried to work it off through exercise.	A strained relationship with food and body	Weighing as a trigger for eating disorders
I did get quite a few comments and then I just started thinking and I compared myself a lot to everyone else around me who was much thinner, and then it was, like, I just wanted to fit in.	When everyone talked about how much they weighed, I started comparing myself to others. It wasn’t really about what was right for me anymore, but about how I measured up to everyone else.	Body and weight comparisons among peers	Increased peer weight talk
I talked to my mom about it, and she kind of responded like, maybe it wasn’t necessary, and she tried to ask if I could at least do it on my own, since the teachers and the school said I had to. If I could just do it by myself instead, because that would have been better, but it still wasn’t possible because it would have taken too much time.	I didn’t feel like I had a choice. Even when I said I didn’t want to, I was told it was just something everyone had to do. I was never given a chance to have any say in how it was done. They just told us we had to get weighed, and I went along with it.	No opportunity to choose or influence how the weighing was carried out	Weighing routines can strengthen or undermine pupils’ autonomy
There hasn’t been any form of follow-up. I was told there would be follow-up, but I haven’t received any. But it might just be a mistake or something I said that she misunderstood or assumed incorrectly.	After I was told my weight wasn’t where it should be, I was supposed to receive some follow-up, but it never happened. I was left feeling uncertain, with a lot of questions I had to figure out on my own.	Absence of expected weight follow-up	Left without support after weighing

### Ethical Considerations

This study was approved by the Norwegian Agency for Shared Services in Education and Research (Project No. 770022), and was guided by the Declaration of Helsinki (World Medical Association, 2013) and the General Data Protection Regulation (European Data Protection Board, 2021). Before participating, adolescents received both oral and written information about the study’s purpose, confidentiality, and the voluntary nature of participation, including the right to withdraw at any time without consequences. All participants provided written informed consent prior to the interviews.

In Norway, individuals aged 16 years and above are considered legally competent to provide informed consent for participation in research (NREC, 2025). Because this study involved qualitative interviews without physical intervention or clinical procedures, participants aged 16 years were considered competent to provide informed consent independently; therefore, parental consent was not required.

## Findings

This section presents the findings from interviews with adolescents regarding their experiences with school-based weighing. The analysis revealed gendered patterns in emotional responses to the weighing process and to weight-related feedback. While some participants described the experience as neutral or informative, others reported increased body dissatisfaction, risky eating behaviors, and experiences of weight-related stigma. Participants also commented on the practical aspects of weighing procedures. The findings are organized into five interrelated themes: (1) neutral reactions to heightened body awareness, (2) weighing as a trigger for eating disorders, (3) increased peer weight talk, (4) weighing routines that can strengthen or undermine pupils’ autonomy, and (5) left without support after weighing.

### From Neutral Reactions to Heightened Body Awareness

Although weighing sometimes evoked discomfort, boys who consistently received normal-weight feedback described it as a neutral or informative experience. They regarded weighing as a routine procedure and a practical component of health monitoring. For these boys, the measurement provided insight into physical development and was viewed as an opportunity for others to receive support if needed.

It’s just, like, in general, to know for yourself whether you are underweight or overweight or in between, so that’s why I think it’s important (Participant 11, boy, 16).

Among some boys who received feedback indicating that they were overweight or underweight described experiencing both discomfort and motivation. One boy explained that being told he was slightly overweight in primary school reduced his confidence, yet it became a turning point that motivated lifestyle changes. When he eventually received normal-weight feedback in lower secondary school, it became a source of pride. Another boy who was classified as underweight described feeling insecure about his body size and later feeling relieved when his weight increased. For these boys, discomfort stemmed from the confronting nature of the feedback, which elicited feelings of shame regarding one’s body size or lifestyle habits; they also emphasized that weighing in school was important and could encourage self-reflection.

Hearing high numbers for my age and being told, ‘You are overweight’ was the last push I needed to make a change. I thought, ‘Well, am I really overweight? I should do something about my body and health.’ I felt guilty for being so big that I could not take care of myself (Participant 12, boy, 16).

Subsequently, girls did not describe such ambivalence. For girls, feedback indicating weight deviation (underweight or overweight) was experienced as unnecessary and unhelpful. This is related to the sense that once it is confirmed that something is wrong, they become more concerned that others might notice perceived flaws in their bodies. The feedback indicating weight deviation was described as contributing to the sense that weight defines self-worth. It also challenged the message often conveyed to children that they are good enough.

Girls who were classified as having normal weight reported discomfort related to the act of being weighed, suggesting that the procedure itself, regardless of the outcome, could increase body awareness through increased attention to weight. One girl described how school-based weighing made her conscious about not gaining weight:

It made me aware of what I weighed, and then you don’t want to gain weight, right. At least in my case. It kind of makes you think more about what you eat, or things like that (Participant 6, girl, 16).

### Weighing as a Trigger for Eating Disorders

Among girls, weighing was described as triggering or exacerbating struggles with food and body image. Narratives indicated a shift from general insecurity to concrete behavioral changes, including food restriction, compulsive physical activity, and binge eating. Three of the eight girls explicitly linked school weighing to the onset of clinically diagnosed eating disorders.

Immediately after I weighed myself at school, I got slightly obsessed with the numbers and my weight. I tried to exercise and eat less, but that just led to binge eating because I got upset. Eventually, I stopped eating together. [. . .] It all came from my weight and the fact that I had to weigh myself and felt different (Participant 14, girl, 16).

Girls also described difficulty interpreting the numbers on the scale, particularly when they lacked prior experience with self-weighing. Peer comparisons overshadowed individual growth assessments, and feedback indicating “normal” or “underweight” could be experienced as threatening when contrasted with classmates’ weights. Heightened focus on weight after school-based weighing sometimes led to increased self-weighing at home.

I saw a higher number while the PHN said I was underweight. I heard my friends’ weights but didn’t think about the fact that they were smaller than me, and that made me feel really bad. [. . .] I kept thinking: where were all those kilos? Where on my body were they? I figured it was around my stomach, and I wanted to change that [. . .] I stopped eating and ended up being hospitalized (Participant 8, girl, 17).

### Increased Peer Weight Talk

School-based weighing inevitably became a topic of discussion among peers. Pupils reported being asked directly, and repeatedly, about their weight immediately after the measurement. Regardless of gender, this created social pressure to disclose weight. Some participants refused to share their weight, believing it to be private. Others disclosed their weight despite discomfort to avoid being judged, conform to perceived social norms, prevent being perceived as “hiding something”, or control the assumptions peers might make. Participants described adjusting the reported number before sharing as a strategy to avoid teasing, reduce judgment, and maintain privacy.

I felt a bit of pressure to share, because everyone else did. I felt like, if I did not, it was like what are you hiding? I felt like changing the number a bit, taking off a couple of kilos instead of saying my real weight. (Participant 14, girl, 16).

Boys described a wide spectrum of responses to weight-related comments, ranging from perceiving them as harmless banter to experiencing them as hurtful. Teasing within close friend groups was often interpreted as playful, but at the same time comments became hurtful when coming from peers outside that circle.

It was like, hey fatty, you’re chubby. They used it in a buddy manner and not personally. But when someone you do not talk to say it, it feels completely different (Participant 1, boy, 17)

While boys described a wide spectrum of responses to weight-related comments, girls’ accounts revealed clearer patterns of discomfort. Narratives included supportive remarks among friend groups, emphasizing that weight did not matter and that one should not be concerned about it. However, such comments could fade during conversations that reinforce expectations of losing weight. Feelings of discomfort could arise from hearing others’ weight and comparing it with their own, and from listening to peers who speak negatively about their bodies or express a desire to become thinner. Girls also stated that boys were not hesitant to make blunt remarks or express strong opinions regarding their weight.

I mean, they were really focused on it, and some were like: no, it’s okay, everyone’s equally valuable. And then, some were like, are you joining the run later? Because I need to lose five kilos before summer [. . .] eighth-grade boys were like: what the hell, you weigh more than me, you fat cow (Participant 15, girl, 16).

Prior experiences of bullying and the pressure to share weight resulted in a strong anticipatory fear prior to school weighing. Peer-questioning expectations, shaped by earlier stigma and comments, contribute to feelings of vulnerability and avoidance. One pupil described how fear of renewed judgement led her to harm herself in an attempt to avoid being weighed at school:

I started dreading it, knowing what would come after. They would say things like I was fat and that I should kill myself [. . .] I tried to make myself sick on purpose. I even tried throwing it up, thinking I would be excused by school (Participant 4, girl, 18).

### Weighing Routines Can Strengthen or Undermine Pupils’ Autonomy

Participants’ experiences exhibited that routines during the weighing situation could either support or undermine their autonomy and well-being. In accounts where participants were asked whether there was anything they would like to change about how weighing was conducted, the pupils noted that they would have appreciated a broader conversation about other aspects of health, enabling the PHN to get to know them better and fostering a greater sense of trust. The pupils also expressed a wish for the PHN to ask how they felt about being weighed. When weighing was embedded within a broader health dialogue that included questions about school, home life, and well-being, it was described as appreciated, though these accounts primarily came from pupils who received normal-weight feedback.

The descriptions highlighted how limited information prior to weighing could create uncertainty about the purpose of the procedure, how the information would be used, and who would have access to it. For instance, one girl recalled feeling anxious afterwards, worrying that her teacher, principal, or other school staff might have been informed about her weight. A lack of information prior to weighing could create the sense that this was simply something everyone was expected to undergo, without the option to raise objections. During the interviews, pupils emphasized that they would have appreciated the opportunity to consent to or decline being weighed, noting that weight can be a sensitive topic and has the potential to cause mental stress afterwards. Participants appreciated the approach in which they were given the option to consent to or influence aspects of the weighing situation. For instance, one girl preferred not to know her own weight and declined to discuss it afterwards. When voluntariness was not communicated, pupils sometimes felt coerced into participating in an activity that later caused significant emotional distress.

She did not explain why we were being weighed; she never said we could say no. I felt like I had to do it because no one told me that it was a choice. (..) I have never liked being weighed because I know that I hyperfixate on it if I know what it says there and what the number is (Participant 9, girl, 16).

Privacy was a central concern. Pupils described being weighed in groups or queue-based systems that compromised confidentiality, particularly when weights were announced aloud. Pupils had no influence over group composition, and exposure to peers heightened discomfort. Requests for individual weighing were declined for efficiency, reducing pupils’ sense of autonomy.

You don’t need to announce that someone weighs xx kilos in front of a group of five boys. [. . .] It wasn’t exactly great when people started sharing it (Participant 15, girl, 16).

Queue-based weighing offered more privacy but still created discomfort. Waiting in groups outside the PHN office fostered weight-related conversations and speculation. Pupils reported rushing through the consultation to avoid attracting attention, even when they had concerns they wanted to discuss.

If you’re the one who takes longer than the others, people start asking why [. . .] I’d probably just want to finish quickly and not draw attention to myself (Participant 12, boy, 16).

### Left Without Support After Weighing

Experiences with follow-up varied significantly, influencing how pupils perceived the PHN. When pupils were informed that their weight deviated from expected growth patterns, they anticipated further contact. However, follow-up was not always provided, leading to confusion about the purpose of weighing and reduced trust in school health services.

I was actually told by the PHN that there would be a follow-up later on. She said I was supposed to have a follow-up every six months. But I have not received any follow-up, either from the doctor, the PHN, or anyone else (Participant 12, boy, 16).

The communication style of PHNs played a crucial role in shaping emotional responses in follow-up conversations with the PHN. One boy described how hearing terms such as *overweight*, *obese*, and *fat* were used to describe his weight. He found *overweight* acceptable but perceived *obesity* and *fat* as judgmental and distancing, causing shock and loss of trust.

Questions about eating habits and physical activity were sometimes perceived as stigmatizing, particularly when they did not reflect pupils’ lifestyles. Advice about healthy eating could feel top-down and disconnected from pupils’ limited influence on family food choices, which are largely determined by parents.

I asked, like, how do you expect me to do this? and she just said I had to talk to my parents. [. . .] If she had listened more to how I felt, maybe it would have been easier to adapt it for me. [. . .] she could have been a bit gentler when bringing up those things. It’s a sensitive topic for many (Participant 7, boy, 17)

Despite these concerns, a few narratives described positive encounters in which PHNs communicated weight-related information sensitively and reassuringly. A neutral tone and messages emphasizing that weight deviations could resolve naturally through growth helped reduce anxiety. In these cases, pupils reported that one conversation was sufficient and that they did not receive any other follow-up.

In the absence of follow-ups, pupils could turn to digital platforms for guidance. However, social media content was described as unreliable and often harmful, promoting unrealistic ideals and dangerous strategies. One girl described relying on online advice at a young age, which led to destructive behaviors.

They don’t really have realistic videos, but that’s what I believed when I was little. [. . .] It was about fasting. That’s something adults can do, but kids shouldn’t. [. . .] I should have received better help with how to do it more healthily, instead of just being told I was overweight and had to deal with it myself as a nine-year-old (Participant 14, girl, 16)

## Discussion

This study demonstrates that retrospective reflections from adolescents provide rich insights into how school-based weighing is experienced and interpreted over time. Although BMI measurements serve important population-level surveillance objectives, producing internationally comparable data and informing policy responses (WHO, 2016, 2017), our findings reveal a complex interplay between these objectives and the individual psychological consequences of weighing in school. Participants reported a broad spectrum of responses, from neutral or practical perspectives that framed weighing as informative to experiences marked by increased body dissatisfaction, social distress, and in some cases, the emergence of disordered eating. Recognizing both positive and negative experiences has important implications for school health services and for how PHNs conduct weight assessments to better support health promotion.

In this study, there were accounts in which pupils expressed a neutral or curious attitude toward school-based weighing, including perceiving potential benefits of receiving feedback on weight development. This finding is consistent with previous quantitative research showing that a substantial proportion of pupils report little or no discomfort related to school weighing (Altman et al., 2022; Drilen et al., 2024). This perspective aligns with the public-health aim of identifying and guiding individuals who may be experiencing weight-related challenges (The Norwegian Directorate of Health, 2017).

At the same time, several accounts in this study conflicted with health-promoting aims. Receiving a numerical weight often heightened body awareness and could evoke body dissatisfaction regardless of the objective weight category. Similar observations have been reported by Nnyanzi (2016) and Jessen et al. (2023), suggesting that school-based weighing may increase weight preoccupation and oversensitivity among pupils.

A particularly concerning finding was participants perceived a connection between school-based weighing and the onset of eating disorders. Although our qualitative data do not establish causality, these narratives underscore the need for ethical reflection and further investigation into the psychological consequences of school weighing. These concerns echo reports from PHNs (Helseth et al., 2017) and from reviews that highlight potential harms of weight-focused practices in schools (Crawford et al., 2011; Ikeda et al., 2006; Ramos Salas, 2015; Thompson & Madsen, 2017). In contrast, Madsen et al. (2021) found in a randomized controlled trial that school-based weighing was associated with a decrease in unhealthy weight control behaviors; however, the study also reported a decline in weight satisfaction among pupils who underwent BMI screening. Similarly, Gee (2015), in an observational study, examined the effects of continued BMI screening among pupils aged 15 to 18 years and found no significant changes in dietary or exercise behavior.

This study also demonstrates that weight measurements rarely ended at the consultation door. This is in line with prior research documenting increased weight-related talk among peers following weighing in schools (Jessen et al., 2023; Madsen et al., 2021). A gendered pattern emerged in which girls described increased pressure from peer comparisons and more sustained negative effects on body image; boys more often framed teasing as banter, although their accounts also included instances of hurtful comments. Schools are regarded as suitable settings for weighing pupils because of their convenient access to children and adolescents (WHO, 2017). Simultaneously, peer opinions and school environment have been identified as powerful influences on how young individuals perceive their body image (Tort-Nasarre et al., 2021; Zanlorenci et al., 2024).

Narratives reflected experiences consistent with weight-related stigma or bullying, which could lead to anxiety, avoidance of school, and harmful coping strategies. Weight-related stigma in healthcare settings has been associated with lower self-esteem, increased body dissatisfaction, and behaviors that undermine health (Puhl & Suh, 2015; Rubino et al., 2020). Therefore, psychosocial risks such as stigmatization, discrimination, and social harm should be considered when implementing school-based weight assessments (Breda et al., 2021; WHO, 2016). PHNs, whose role includes promoting physical and mental health (The Norwegian Directorate of Health, 2021), are well placed to reduce harm by using stigma-free, person-centered communication and by addressing how body-related comments affect pupils well-being.

Participants’ experiences of feedback and follow-up varied considerably. Feedback was described as intrusive and stigmatizing by some participants, particularly those who were told they were overweight, whereas others recalled respectful, normalizing conversations. Existing literature documents that individuals with higher body weight can encounter stigma in healthcare settings, which may discourage future help-seeking (Rubino et al., 2020). A systematic review of weight-related terminology recommends that healthcare providers ask patients how they prefer to discuss weight and obtain permission before raising the topic (Puhl, 2020). PHNs in qualitative studies have similarly called for clearer guidelines and additional training in communication about weight (Helseth et al., 2017; Nygaard & Øen, 2024). A joint international consensus for ending obesity stigma recommends that healthcare professionals working in this field acquire and further develop their knowledge as well as skills for stigma-free practices (Rubino et al., 2020). Our findings support these recommendations: explicitly inviting pupils to express how they feel about weighing, explaining the purpose and voluntariness of measurements, and tailoring conversations to each pupil’s context may reduce unintended psychological harm.

Organizational factors also influenced experiences. Similar to a prior study (Igler et al., 2022), the findings of this study point to several organizational shortcomings in the implementation of BMI measurements in schools. Pupils reported receiving limited information about the purpose of weighing and who would have access to the results. It was also described as not knowing whether participation was voluntary. This lack of communication appears to undermine pupils’ perceptions of weighing themselves as a health-promoting initiative, creating a sense of detachment rather than engagement. The national guidelines in Norway recommend that measurements be conducted at the end of an extended health consultation (The Norwegian Directorate of Health, 2017). This consultation can provide an opportunity to inform pupils about the procedure and ensure that the situation is handled sensitively and on the individual’s terms.

A recurrent concern was the absence of promised follow-up. Pupils described brief or impersonal conversations that left them feeling solely responsible for change, despite limited agency over family food environments and other structural factors. In such cases, pupils could turn to social media for guidance; however, online content could be unreliable and promote harmful practices. This highlights a need for clear referral pathways, adequate resources for follow-up, and developmentally appropriate support when weight deviations are identified (Helseth et al., 2017; Nordstrand et al., 2016; Nygaard & Øen, 2024). It should be noted that this study is based on pupils’ accounts. According to national guidelines (The Norwegian Directorate of Health, 2017), information about weight and offers of follow-up is primarily directed toward parents. Consequently, this study does not provide insight into parental involvement. The lack of effective interventions for BMI measurements and obesity prevention has been discussed in several research articles as well as commentaries (Jessen et al., 2023; Madsen et al., 2021; Ramos Salas, 2015; Thompson & Madsen, 2017) and further underscores the importance of carefully weighing potential benefits against psychological risks.

### Study Limitations

Several methodological limitations should be acknowledged in relation to Lincoln and Guba’s (1985) criteria for trustworthiness in qualitative research, namely: credibility, transferability, dependability, and confirmability.

First, retrospective accounts may be affected by recall bias or reconstructive memory (Neusar, 2014); however, temporal distance can also allow more reflective and nuanced narratives. Second, the researchers’ roles as PHNs may have influenced participants’ accounts, either by facilitating trust or by shaping responses. We attempted to mitigate this through a neutral, participant-led interview approach and reflexive analytic practices. Third, recruitment was voluntary and may have introduced self-selection bias (Polit & Beck, 2021); individuals with particularly strong experiences could be overrepresented. Nevertheless, the purposive sampling strategy captured variation in age, sex, and school context.

Fourth, the STC method (Malterud, 2012) involves condensation that can decontextualize data; to address this, we recontextualized findings and used the NVivo software to maintain an audit trail and enhance dependability. Finally, researchers’ pre-understandings may have influenced interpretation; collaborative analysis, reflexive memoring, and transparent documentation were used to limit bias and strengthen confirmability.

## Conclusions and Clinical Implications

This study represents an initial step toward understanding how pupils experience and interpret school-based weighing. Although BMI measurements are implemented with health-promoting intentions, the findings indicate that this practice may undermine those aims, particularly when psychological, social, and organizational factors are considered. The pupils’ accounts offer valuable insight into how PHNs can approach weighing in ways that better align with health-promoting principles.

Psychosocial consequences such as body dissatisfaction, peer comparison, stigma, and social pressure are important risks associated with school-based weighing. Ensuring privacy, clearly communicating the purpose of weighing, and emphasizing voluntary participation are essential. Health consultations should allow space for dialogue, enabling pupils to ask questions and express concerns. PHNs should also have opportunities to acquire training in stigma-free language and practices. Scheduling health consultations for large groups on the same day or within a short timeframe may increase peer discussions about weight. We therefore suggest organizing consultations so that pupils are seen individually and across the school year to reduce opportunities for weight-related comparisons, bullying, and social pressure.

Although this study cannot determine the psychological cost of school-based weighing, the narratives included instances in which weighing at school was described as a trigger for disordered eating patterns, warranting careful ethical consideration. These findings, combined with other psychosocial consequences, contribute to the broader debate on whether school-based weighting should be conducted at all.

## Supplemental Material

sj-docx-1-gqn-10.1177_23333936261435414 – Supplemental material for Adolescents’ Experiences with Being Weighed at School: A Qualitative Interview Study in Norway Supplemental material, sj-docx-1-gqn-10.1177_23333936261435414 for Adolescents’ Experiences with Being Weighed at School: A Qualitative Interview Study in Norway by Marit Kjesbo Risøy and Maria Gangdal Gunnarskog in Global Qualitative Nursing Research
