# Supplementary material for: Adolescents’ Experiences with Being Weighed at School: A Qualitative Interview Study in Norway
Source: Glob Qual Nurs Res. 2026 Mar 27;13:23333936261435414. doi: 10.1177/23333936261435414 (PMC13033862; doi:10.1177/23333936261435414)
Supplement: sj-docx-1-gqn-10.1177_23333936261435414 – Supplemental material for Adolescents’ Experiences with Being Weighed at School: A Qualitative Interview Study in Norway [file sj-docx-1-gqn-10.1177_23333936261435414.docx]

Interview guide

The interview guide was originally developed and used in Norwegian. The version provided here is translated into English for review purposes.

Opening question: Could you briefly tell me a little about yourself?

Age, and which study program are you enrolled in?

How are things for you at school?

How are things for you in your free time?

| **Main question** | **Possible follow-up questions** |
| --- | --- |
| 1. How did you experience being weighed by the school nurse? | - Have you ever had a difficult experience with weighing at school, and can you tell me about it? - Have you had any positive experiences with weighing at school, and can you tell me about it? - What did you do in the situation? |
| 2. Have you talked with anyone else about being weighed at school? For example, friends or family? What did they say or think about it? | - How was that for you? - Did you feel pressure to share your weight result with classmates? If yes, how did that pressure feel? |
| 3. How do you think the feelings connected to being weighed affect your self-image, self-esteem, and confidence? | - Do you see your body differently after being weighed? If yes, in what way? - Have you made any changes in your life after being weighed? Positive or negative? How was that for you? - Has school-based weighing influenced your view of food and diet? If yes, in what way? - Has school-based weighing influenced your exercise habits or level of physical activity? If yes, in what way? |
| Why do you think pupils are weighed at school? | - What do you think about the school weighing pupils? - Is there anything you would like to change about how weighing is carried out in school? - If you could choose, would you accept being weighed at school today? Why/why not? |
| How did you experience the school nurse during the conversation about weighing? | - How did the school nurse talk with you about weight? - How did the school nurse prepare you for the weighing? - What information did the school nurse give you about why you were being weighed? - In what way did you feel your opinions or concerns were heard and taken seriously? - If there was something you did not want to do, were you allowed to refrain? - Is there anything about the situation that could have been done differently to make you feel better? - How did you feel your privacy was respected in the conversation and during the weighing? - How do you think the conversation about weighing affected your trust in the school nurse? Did you trust the nurse more or less after this? |
